# Does Rocuroinum Dose Adjusted Due to Lean Body Weight Provide Adequate Intubation Conditions?: A Prospective Observational Study

**DOI:** 10.1155/2022/6840960

**Published:** 2022-10-05

**Authors:** Duygu Demiroz, Yusuf Ziya Colak, Sumeyye Koc Iclek, Mehmet Ali Erdogan, Neslihan Altunkaya Yagci, Mahmut Durmus, Nurcın Gulhas

**Affiliations:** ^1^Inonu University, School of Medicine, Department of Anesthesiology and Reanimation, Malatya, Turkey; ^2^Bezmialem Foundation University Medical Faculty Hospital, Department of Anesthesiology and Reanimation, Istanbul, Turkey

## Abstract

**Methods:**

This is a prospective, observational study. Patients between the ages of 18 and 65 with BMI of 18.5–34.9, who are expected to be under general anesthesia for less than 6 hours, were divided into 3 groups according to their BMI (Group 1 BMI = 18.5–24.9, Group 2 BMI = 25–29.9, Group 3 BMI = 30–34.9). These groups were randomly divided into 2 subgroups: Groups LBW; 1 LBW, 2 LBW, and 3 LBW were given rocuronium intubation dosages based on their LBW while control groups; 1K, 2K, and 3K were given 0.6 mg/kg rocuronium according to their total body weight. The data on the duration of action of rocuronium and its effects on the endotracheal intubation conditions were evaluated.

**Results:**

In Group 1, *T*1 time was found to be significantly longer (*p*=0.001). Intubation score and the use of additional rocuronium dose were found to be significantly higher in Group 1 LBW than in Group 1K (*p*=0.001). In Group 1, an additional rocuronium dose was needed to achieve optimal intubation conditions for subgroup 1 LBW. Rocuronium duration of action was found to be significantly longer in control groups 2 and 3, that received TBW-based dosage.

**Conclusion:**

In adult patients with a BMI of 18.5 and 24.9 BMI, we report optimal intubation conditions with the LBW-adjusted rocuronium dosage. This trial is registered with NCT05476952.

## 1. Introduction

Rocuronium is a hydrophilic, nondepolarizing neuromuscular blocker widely used to provide muscle relaxation during surgery to facilitate ventilation of the lungs in elective or emergencies. Because the distribution of hydrophilic drugs is limited to lean tissue only, dosing should generally be done using ideal body weight (IBW) or adjusted body weight [1]. Dosing using lean body weight (LBW) has been reported to yield more accurate results compared to total body weight (TBW) [2]. LBW is significantly correlated with cardiac output, which is an important determinant in the early distribution kinetics of drugs. In addition, drug clearance increases proportionally with LBW [3]. Appropriate dosage use of neuromuscular blocking agents (NMBA) is important because of the risk of postoperative residual curarization (PORC). PORC occurs in at least 20% to 40% of patients and is recognized as a common problem in the postanesthetic care unit (PACU) [4]. PORC can cause postoperative pulmonary complications such as aspiration, pneumonia, and atelectasis. The incidence of PORC is related to the duration of action of neuromuscular blocking agents [5].

Most studies on the dosing of neuromuscular blocking agents were performed in obese patients with a BMI of 35 and above. In literature, there was no study on rocuronium doses in patients with a BMI between 18.5 and 35 based on total and lean body weight. The current study aimed to compare the effects of total (TBW) and lean body weight (LBW) adjusted rocuronium doses on optimal intubation conditions in patients with BMI of 18.5 to 35.

## 2. Material and Methods

This prospective randomized study was performed in Turgut Ozal Medical Center between September 2021 and May 2022, after the approval of the Inonu University ethics committee (decision no; 2021/169). (Clinical trials ID; NCT05476952) [6]. Patients aged between 18 and 65 years, with ASA (American Society of Anesthesiologists physical status) ≤ III and BMI of 18.5–34.9. General surgery patients who will undergo abdominal surgery under general anesthesia for which neuromuscular blockade lasting less than 6 hours is required were included in the study. Those with renal, hepatic, neuromuscular, and metabolic diseases; craniotomies, cardiac, thoracic, and large vessel surgeries; and those who did not want to participate in the study were excluded from the study. Written informed consent form was obtained from all patients included to study.

**Figure 1:**
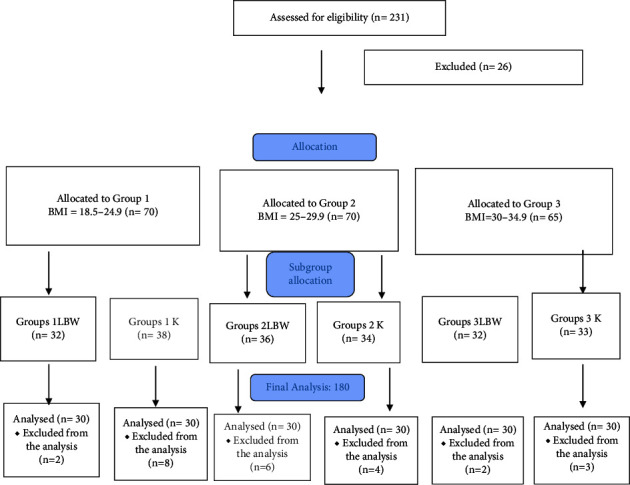
Flowchart.

A total of 205 patients were recruited. 26 patients were excluded from the study. 60 patients from each group were included in the study. Before the surgery, total and lean body weight were calculated using the Tanta-BC 418 device using the Bioelectric Impedance Analysis System, age, sex, height, weight, and BMI of the patients were recorded [7]. Patients with a normal weight between 18.5 and 24.9 BMI were assigned Group 1, overweight patients with a BMI between 25 and 29.9 were assigned Group 2, and patients with a BMI of 30–34.9 in obesity class 1 were placed in Group 3 [8] (Figure 1). The groups were randomly divided into 2 groups among themselves. The patients who were administered rocuronium according to LBW were categorized as “LBW”, and the group number for those administered according to TBW was categorized as “K”. Patients were randomly and equally divided into 2 groups based on a computer-generated list of random numbers placed in opaque sealed envelopes. One group was given 0.6 mg/kg rocuronium on LBW basis (Curon® 50 mg/5 mL [Mustafa Nevzat, Istanbul, Turkey]), and the other group was given 0.6 mg/kg rocuronium on TBW basis.

All patients were routinely monitored by electrocardiogram, pulse oximetry, and noninvasive arterial pressure. For the induction of anesthesia, intravenous (IV) fentanyl 50–100 *μ*g and propofol 2 mg/kg were administered and ventilated with 100% oxygen. After achieving optimal general anesthesia, the ulnar was monitored at the wrist with TOF-Watch® SX (Organon, Swords Co., Dublin, Ireland) by stimulating the nerve every 15 seconds. A bolus rocuronium of 0.6 mg/kg was administered to the LBW subgroup. A similar dosage based on the TBW was administered to the K subgroup. The time from rocuronium administration to the disappearance of TOF responses was measured (*T*1 (second)). All patients were intubated using a Macintosh laryngoscope with an endotracheal tube with internal diameters ranging from 7.5 to 8.0 mm for men and 7.0 to 7.5 mm for women. A single experienced anesthetist evaluated intubation conditions according to the Helbo-Hansen Raulo intubation scoring system (Table 1). An additional dose of 10 mg rocuronium was administered to patients with an intuition score of 9 and above. Anesthesia was maintained with sevoflurane and additional doses of fentanyl. Monitorization with TOF was continued until the initial fasciculation strength (*T*2 = minute) of the adductor pollicis dropped to 25%, and then an additional rocuronium dose of 0.3 mg/kg was administered. At the end of the operation, all anesthetic agents were discontinued, and the patients were ventilated with 100% oxygen. When the TOF value reached 75%, they were de-curarized (neostigmine/atropine) and extubated when the TOF value reached 90%. After extubating, the patients consciousness, activity, respiration, circulation, and peripheral oxygen saturation were evaluated according to the Modified Aldrete Recovery Score at the 1st, 10th, and 30th minute. In addition, adverse effects such as nausea, vomiting, sore throat, laryngospasm, and hoarseness were noted. All patients transferred to postoperative care unit. No patients died during surgery.

### 2.1. Statistical Analysis

The Statistical Package for Social Sciences for Windows version 15 (SPSS Inc., Chicago, IL, USA) was used for statistical analysis. The distribution of the data was tested with the Shapiro–Wilks test. The data were shown as the number of patients, or median and range. The Mann–Whitney U or student's *t*-test was used for comparison of continuous data, according to the distribution of data. The chi-square test was used for the comparison of categorical data. A *p* < 0.05 was considered statistically significant.

In the power analysis, the number of patients in each group was calculated to be at least 46 to evaluate the significant difference in the time between the rocuronium dose according to lean body weight and the dose difference applied according to the actual body weight, until the train of four (TOF) reaches 90% and intubation conditions, with 5% error 80% power.

## 3. Results

Group 1 included patients with BMI 18.5–24.9 normal weight; when Group 1 LBW and 1K were compared, the mean age, gender, height, weight, operation time, and lean body weight of the patients were similar (Table 2). In Group 1, *T*1 time was found to be significantly longer (*p*=0.001). Intubation score and the use of additional rocuronium dose were found to be significantly higher in Group 1 LBW than in Group 1K (*p*=0.001) (Table 3). *T*2 duration was found to be similar between Group 1 LBW and Group 1K. (*p*=0.867) (Table 2).

When the overweight patients—that included patients with BMI 25–29.9 (Group 2)—were evaluated, the mean age, gender, height, weight, operation time, and lean body weight of the patients between Group 2 LBW and 2K showed no significant difference between the two groups (Table 2). *T*1 time was longer in group K but there was no significant difference between groups (*p*=0.085). Intubation score was similar in both groups (*p*=0.115). Intubation times were similar between subgroups (*p*=0.622), and no significant difference was observed between additional rocuronium dose requirements (*p*=0.001). When we evaluated the *T*2 times, it was observed that it was significantly longer in Group 2K compared to Group 2 LBW (*p*=0.042) (Table 3).

When BMI 30–34.9 obesity class 1 patients (Group 3) were evaluated, the mean age was 42.6 and 39.2 for Group 3 LBW and 3K, respectively, and was similar. When gender, height, weight, operation time, and lean body weight were evaluated, no statistically significant difference was observed. There was no significant difference between *T*1 times (*p*=0.578). There was no significant difference between the intubation score (*p*=0.649) and intubation time (*p*=0.474). It was observed that there was no need for additional rocuronium doses in both groups (Table 3). *T*2 time was significantly shorter in Group 3 LBW than in Group K, and the difference was statistically significant (*p*=0.010) (Table 3).

There was no significant difference between the subgroups in the Modified Aldrete Recovery Scoring at the 1st, 10th, and 30th minutes in all the 3 groups. In addition, in terms of nausea, vomiting, sore throat, laryngospasm, and hoarseness, we did not observe any significant differences in any group comparisons. (*p* > 0.05).

## 4. Discussion

NMB agents are widely used in anesthesia induction to provide endotracheal intubation and to provide appropriate muscle relaxation during the operation. There is no consensus in the guidelines about the weight parameter to be used for weight-based dosing of NMBA. Studies on this subject were mostly conducted for patients with a BMI above 35. To the best of our knowledge, there is no study comparing the effects of total (TBW) and lean body weight (LBW) adjusted rocuronium doses on optimal intubation conditions in patients with BMI of 18.5 to 35. We observed that adequate intubation conditions were achieved with LBW-adjusted 0.6 mg/kg rocuronium dose in overweight (BMI of 25–29.9) and obesity class 1 (BMI of 30–34.9) patients. As a secondary result, we observed that the effect time of rocuronium administered according to TBW in overweight and obesity class 1 patients is significantly longer than LBW-adjusted dose.

Rocuronium is a hydrophilic nondepolarizing NMBA, and in practice, rocuronium doses are administered as 0.6 mg·kg as an initial dose and 0.15 mg·kg for additional doses. Since the distribution of hydrophilic drugs is limited to lean tissue, dosing is usually done using ideal body weight or adjusted body weight [1]. Hydrophilic drugs usually have a higher plasma concentration and a lower volume of distribution. Lean body mass is defined as body weight devoid of all fat mass. Dosing using LBW allows for more accurate results compared to total body weight and contributes to approximately 99% of drug clearance [9]. In our study, we calculated the rocuronium dose to be used in intubation according to TBW and lean body weight and administered it at a dose of 0.6 mg/kg. The Tanta-BC 418 is a device that works with the Bioelectrical Impedance Analysis (BIA) method and measures the fat ratio, muscle mass, and lean mass values. The suitability of the cost-effective and portable Tanta-BC 418 for body composition assessment in the adult population was evaluated and highlighted by Vasold et al. [5]. We administered the drug dose using the measurements of the patients' lean body weight calculated by the Tanta-BC 418 device.

In studies conducted on obese patients, (BMI ≥ 40 kg/m^2^) because overdose is more of a concern than inadequate dosing IBW-adjusted dosing had been preferred [7]. Postoperative residual curarization (PORC) which can be due to overdose, may cause postoperative pulmonary complications such as aspiration, pneumonia, and atelectasis. The incidence of postoperative residual curarization is related to the duration of action of neuromuscular blocking agents [10, 11]. It has been shown that excellent or good tracheal intubation conditions are achieved within 60 seconds after administration of a rocuronium dose of 1.2 mg/kg based on LBW in obese patients [12]. In another study, it was emphasized that 0.3 mg/kg given to low-weight patients provided intubation conditions in 18 of 20 patients with optimal conditions [13]. In our study, we observed that the LBW-adjusted rocuronium dose in patients with normal body weight (BMI of 18.5–24.9) did not achieve optimal intubation conditions. However, excellent intubation conditions were achieved in overweight (BMI of 25–29.9) and obesity class 1 (BMI of 20–34.9) patients with lean body weight-adjusted dosage.

Studies on the duration of action of MNDAs have shown that the duration of action of the drugs was similarly prolonged in advanced age and female patients [14, 15]. In our study, patient age and gender ratios were found to be similar in all subgroups, and we observed that they did not affect the duration of action.

In a study conducted on obese patients, intubation conditions, onset time of blockade, and duration of action were evaluated by administering rocuronium at a dose of 1.2 mg/kg according to ideal body weight and lean body weight. It was shown that the duration of action is shorter in the rocuronium dose administered compared to LBW [12]. Fujimoto et al. evaluated the relationship between rocuronium action time and BMI by administering rocuronium at a dose adjusted according to TBW in patients with BMI in the range of 15–30 kg/m^2^ and found a correlation [16]. Similarly, two different studies demonstrated that the duration of action of TBW-adjusted rocuronium was directly proportional to an increase in BMI [17, 18]. In our study, the duration of action of LBW-adjusted rocuronium dose was found to be similarly shorter in patients with normal body weight (BMI of 18.5–24.9). We think that this result is due to the additional doses given to patients who could not achieve adequate intubation conditions. In patients with BMI of 25–29.9 and 30–34.9, it was observed that the duration of action was prolonged in those who received TBW-adjusted dosage.

There are several limitations of our studies. The major limitation of this study is the small number of patients. But power analysis showed that 46 patients for each group was enough, however, in our study each group has 60 patients. The second limitation is being a single-center study.

## 5. Conclusion

In patients with BMI of 18.5–24.9, we achieved faster and optimal intubation conditions with LBW-adjusted rocuronium dose. We cannot report the same results for other BMI groups, and we did not describe any differences in terms of complications. That is why more studies should be warranted to confirm our results.

## Figures and Tables

**Table 1:** Intubating condition score.

Score	1	2	3	4
Laryngoscopy	Easy	Fair	Difficult	Impossible
Vocal cords	Open	Moving	Closing	Closed
Coughing	None	Slight	Moderate	Severe
Jaw relaxation	Complete	Slight	Stiff	Rigid

**Table 2:** Patient data table.

	Group 1 LBW (*n* = 60)	Group 1K (*n* = 60)	*p*-value	Group 2 LBW (*n* = 60)	Group 2K (*n* = 60)	*p*-value	Group 3 LBW (*n* = 60)	Group 3K (*n* = 60)	*p*-value
Age (yrs.)	29.7 (8.7)	29.3 (7.9)	0.975	35.5 (9.5)	35.1 (9.4)	08.40	42.6 (8.5)	39.2 (10)	0.162
Sex (female)	14 (%46.7)	14 (%46.7)	0.602	12 (%40)	11 (%36.7)	0.623	14 (%46.7)	15 (%50)	0.800
Height (cm)	170.4 (8.9)	171.4 (7.6)	0.633	166.9 (7.8)	170.1 (5.9)	0.78	167 (8.3)	165.6 (9.3)	0.543
Weight (kg)	63.5 (9.1)	63.8 (9.1)	0.895	75.8 (8.6)	78.8 (6.2)	0.128	89.1 (8.5)	89.6 (10.1)	0.815
BMI (kg/m^2^)	21.7 (1.6)	21.5 (1.7)	0.792	27.1 (1.5)	27.1 (1.1)	0.906	31.9 (1.4)	32.5 (1.2)	0.051
Operation time (minute)	220.6 (40.4)	212.6 (43.7)	0.855	216.4 (30.2)	210.5 (31.5)	0.139	140.6 (14.3)	153.7 (26.3)	0.101
LBW	48.9 (7.7)	48.1 (7.7)	0.690	50.7 (7.6)	53 (6.1)	0.210	52.7 (4.1)	52.9 (6.1)	0.900

*p* < 0.05 was determined as statistically significant, BMI: body mass index, LBW: lean body weight (LBW), K: control.

**Table 3:** Comparison of the groups in terms of intubation conditions and duration of rocuronium effect.

	Group 1 LBW (*n* = 60)	Group 1K (*n* = 60)	*p*-value	Group 2 LBW (*n* = 60)	Group 2K (*n* = 60)	*p*-value	Group 3 LBW (*n* = 60)	Group 3K (*n* = 60)	*p*-value
*T*1 (second)	173.9 (45.8)	138.7 (30.4)	0.001	138.7 (30.4)	128.2 (11.7)	0.085	132.5 (11.2)	134.3 (13.9)	0.578
Intubation condition	7 (2.8)	4.8 (1.7)	0.001	4.8 (1.7)	4.3 (0.5)	0.115	4.1 (0.3)	4.1 (0.3)	0.649
Intubation time (second)	8.5 (1.2)	8.1 (0.7)	0.003	8.1 (0.7)	8.2 (0.8)	0.622	8.4 (0.8)	8.6 (0.1)	0.474
Total rocuronium dose (mg)	29.4 (4.6)	31 (4.3)	0.149	31 (4.3)	46.5 (4.4)	0.0001	32.1 (2.5)	52.2 (7.4)	0.001
Additional rocuronium dose (mg)	6 (4.9)	1 (3.1)	0.0001	0.1 (0.3)	0	0.0001	0	0	0.001
*T*2 (minute)	30.6 (1.6)	30.7 (1.8)	0.876	23 (1.7)	34.7 (2.8)	0.042	21.9 (1.7)	41.2 (1.7)	0.010

*p* < 0.05 was determined as statistically significant, BMI: body mass index, LBW: lean body weight (LBW), K: control.

## Data Availability

The data used to support the findings of this study are available from the corresponding author upon request.
